# Clinical trials in pediatric ALS: a TRICALS feasibility study

**DOI:** 10.1080/21678421.2021.2024856

**Published:** 2022-02-16

**Authors:** Tessa Kliest, Ruben P.A. Van Eijk, Ammar Al-Chalabi, Alberto Albanese, Peter M. Andersen, Maria Del Mar Amador, Geir BrÅthen, Veronique Brunaud-Danel, Lev Brylev, William Camu, Mamede De Carvalho, Cristina Cereda, Hakan Cetin, Delia Chaverri, Adriano Chiò, Philippe Corcia, Philippe Couratier, Fabiola De Marchi, Claude Desnuelle, Michael A. Van Es, JesÚs Esteban, Massimiliano Filosto, Alberto GarcÍa Redondo, Julian Grosskreutz, Clemens O. Hanemann, Trygve HolmØy, Helle HØyer, Caroline Ingre, Blaz Koritnik, Magdalena Kuzma-Kozakiewicz, Thomas Lambert, Peter N. Leigh, Christian Lunetta, Jessica Mandrioli, Christopher J. Mcdermott, Thomas Meyer, Jesus S. Mora, Susanne Petri, MÓnica Povedano, Evy Reviers, Nilo Riva, Kit C.B. Roes, Miguel Á. Rubio, FranÇois Salachas, Stayko Sarafov, Gianni SorarÙ, Zorica Stevic, Kirsten Svenstrup, Anette Torvin MØller, Martin R. Turner, Philip Van Damme, Lucie A.G. Van Leeuwen, Luis Varona, Juan F. VÁzquez Costa, Markus Weber, Orla Hardiman, Leonard H. Van Den Berg

**Affiliations:** 1 Department of Neurology, UMC Utrecht Brain Centre, University Medical Centre Utrecht, Utrecht, the Netherlands; 2 Biostatistics & Research Support, Julius Centre for Health Sciences and Primary Care, University Medical Centre Utrecht, Utrecht, the Netherlands; 3 Department of Basic and Clinical Neuroscience, King's College London, Maurice Wohl Clinical Neuroscience Institute, London, UK; 4 Department of Neurology, King’s College Hospital, London, UK; 5 IRCCS Humanitas Research Hospital Rozzano, Milan, Italy; 6 Department of Clinical Sciences, Neurosciences, Umeå University, Umeå, Sweden; 7 Département de Neurologie, Centre de référence SLA Ile de France; 8 Hôpital de la Pitié Salpêtrière, AP-HP, Paris, France; 9 Department of Neurology, University Hospital of Trondheim, Trondheim, Norway; 10 Department of Neuromedicine and Movement Science, Norwegian University of Science and Technology, Trondheim, Norway; 11 ALS centre, CHU, University of Lille Nord de France, Lille, France; 12 Bujanov Moscow City Clinical Hospital, Moscow, Russian Federation; 13 Moscow Research and Clinical Center for Neuropsychiatry of the Healthcare Department, Moscow, Russian Federation; 14 ALS Centre CHU Gui de Chauliac, University of Montpellier, Montpellier, France; 15 Institute of Physiology-Instituto de Medicina Molecular, Faculty of Medicine, University of Lisbon, Lisbon, Portugal; 16 Department of Neurosciences and Mental Health, H Santa Maria-CHLN, Lisbon, Portugal; 17 Regional Newborn Screening Laboratory, Vittore Buzzi Children's Hospital-University of Milan, Italy; 18 Department of Neurology, Medical University of Vienna, Vienna, Austria; 19 Neurology Service, Hospital Universitario La Paz, Madrid, Spain; 20 'Rita Levi Montalcini' Department of Neuroscience, ALS Centre, University of Torino, Turin, Italy; 21 Azienda Ospedaliera Città della Salute e della Scienza, Turin, Italy; 22 Centre Constitutif SLA, CHRU de Tours - Fédération des centres SLA Tours-Limoges, LitORALS, Tours, France; 23 Centre Constitutif de reference SLA-Fédération Tours-Limoges, CHU de Limoges, Limoges, France; 24 "Maggiore della Carità" University Hospital, Novara, Italy; 25 Hôpital Pasteur 2 – CHU de Nice, Nice, France; 26 ALS Research Lab - ALS Unit, Instituto de Investigación Sanitaria Hospital 12 de Octubre "i + 12", CIBERER, Madrid, Spain; 27 Department of Clinical and Experimental Sciences, University of Brescia; NeMO-Brescia Clinical Center for Neuromuscular Diseases, Brescia, Italy; 28 Precision Neurology, Dept. of Neurology, Lübeck University Hospital, Lübeck, Germany; 29 University of Plymouth, Peninsula Schools of Medicine and Dentistry, Plymouth, UK; 30 Department of Neurology, Akershus University Hospital, Lørenskog, Norway; 31 Institute of Clinical Medicine, University of Oslo, Oslo, Norway; 32 Department of Medical Genetics, Telemark Hospital, Skien, Norway; 33 Department of Neurology, Karolinska University Hospital, Stockholm, Sweden; 34 Department of Clinical Neuroscience, Karolinska Institutet, Stockholm, Sweden; 35 Institute of Clinical Neurophysiology, University Medical Centre Ljubljana, Ljubljana, Slovenia; 36 Department of Neurology, Medical University of Warsaw, Warsaw, Poland; 37 Department of Neurology, Royal Stoke University Hospital, Stoke, United Kingdom; 38 Department of Neuroscience, Brighton and Sussex Medical School, Trafford Centre for Biomedical Research, University of Sussex, Brighton, UK; 39 NEMO Clinical Center, Serena Onlus Foundation, Milan, Italy; 40 NEMO LAB, Milan, Italy; 41 Department of Biomedical, Metabolic and Neural Sciences, Center for Neuroscience and Neurotechnology, University of Modena and Reggio Emilia, Modena, Italy; 42 Department of Neuroscience, St. Agostino Estense Hospital, Azienda Ospedaliero Universitaria di Modena, Modena, Italy; 43 Department of Neuroscience, University of Sheffield, Sheffield Institute for Translational Neuroscience, Sheffield, United Kingdom; 44 ALS Outpatient Department, Charité – Universitatsmedizin Berlin, Berlin, Germany; 45 ALS Unit/Neurology, Hospital San Rafael, Madrid, Spain; 46 Department of Neurology, Hannover Medical School, Hannover, Germany; 47 Functional Unit of Amyotrophic Lateral Sclerosis (UFELA), Service of Neurology, Bellvitge University Hospital, Hospitalet de Llobregat, Spain; 48 European Organization for Professionals and Patients with ALS (EUpALS) & ALS Liga Belgium, Leuven, Belgium; 49 Department of Neurology, Experimental Neuropathology Unit, Institute of Experimental Neurology, Division of Neuroscience, San Raffaele Scientific Institute, Milan, Italy; 50 Department of Health Evidence, Section Biostatistics, Radboud University Medical Centre Nijmegen, Nijmegen, the Netherlands; 51 Neuromuscular Unit, Department of Neurology, Hospital del Mar, Barcelona, Spain; 52 Instituto Hospital del Mar de Investivaciones Médicas (IMIM), Barcelona, Spain; 53 Clinic of General Neurology, Medical University Sofia, University Hospital Alexandrovska, Sofia, Bulgaria; 54 Department of Neurosciences, University of Padova, Padova, Italy; 55 Clinic of Neurology, Clinical Center of Serbia, School of Medicine, University of Belgrade, Belgrade, Serbia; 56 Department of Neurology, Bispebjerg-Frederiksberg Hospital and Rigshospitalet, University Hospital of Copenhagen, Denmark; 57 Department of Neurology, Aarhus University Hospital, Aarhus, Denmark; 58 Nuffield Department of Clinical Neurosciences, University of Oxford, Oxford, UK; 59 Department of Neurosciences, Laboratory for Neurobiology, KU Leuven and Centre for Brain & Disease Research, VIB, Leuven Brain Institute, Leuven, Belgium; 60 Department of Neurology, University Hospitals Leuven, Leuven, Belgium; 61 Department of Neurology, Basurto University Hospital, Vizcaya, Spain; 62 ALS Unit and Neuromuscular Disease Unit, Department of Neurology, Hospital La Fe, Valencia, Spain; 63 Neuromoscular Disease Unit/ALS Clinic, Cantonal Hospital St. Gallen, St. Gallen, Switzerland; 64 Academic Unit of Neurology Trinity College Dublin Ireland, Dublin, Ireland

**Keywords:** Pediatric amyotrophic lateral sclerosis, clinical trial, pediatric investigation plan, clinical trials, ethics, therapy

## Abstract

*Background:* Pediatric investigation plans (PIPs) describe how adult drugs can be studied in children. In 2015, PIPs for Amyotrophic Lateral Sclerosis (ALS) became mandatory for European marketing-authorization of adult treatments, unless a waiver is granted by the European Medicines Agency (EMA).

*Objective:* To assess the feasibility of clinical studies on the effect of therapy in children (<18 years) with ALS in Europe.

*Methods:* The EMA database was searched for submitted PIPs in ALS. A questionnaire was sent to 58 European ALS centers to collect the prevalence of pediatric ALS during the past ten years, the recruitment potential for future pediatric trials, and opinions of ALS experts concerning a waiver for ALS.

*Results:* Four PIPs were identified; two were waived and two are planned for the future. In total, 49 (84.5%) centers responded to the questionnaire. The diagnosis of 44,858 patients with ALS was reported by 46 sites; 39 of the patients had an onset < 18 years (prevalence of 0.008 cases per 100,000 or 0.087% of all diagnosed patients). The estimated recruitment potential (47 sites) was 26 pediatric patients within five years. A majority of ALS experts (75.5%) recommend a waiver should apply for ALS due to the low prevalence of pediatric ALS.

*Conclusions:* ALS with an onset before 18 years is extremely rare and may be a distinct entity from adult ALS. Conducting studies on the effect of disease-modifying therapy in pediatric ALS may involve lengthy recruitment periods, high costs, ethical/legal implications, challenges in trial design and limited information.

## Introduction

In 2007, the clinical drug development pathway for adult populations changed when the Pediatric Regulation came into force. The aim of the Pediatric Regulation is to provide better medicines for patients below the age of 18 years in the European Union (1). To encourage the development of new drugs for children, marketing-authorization applications of drugs for adults must contain an approved Pediatric Investigation Plan (PIP), or clinical trial data of the pediatric population. This mandatory and additional clinical investigation plan describes how safety and/or efficacy of an adult treatment can be studied in the pediatric population. The Pediatric Committee (PDCO), a committee of the European Medicines Agency (EMA), is responsible for the evaluation and approval of PIPs. When a PIP is approved, pharmaceutical- or biotech companies are required to conduct a study in compliance with this PIP to obtain necessary data for the authorization of a medicine for the pediatric population. A company can be exempted from conducting studies in children if the PDCO grants a waiver. Waivers are granted when the disease only occurs in adults. If a disease does occur in children, a company may request a waiver when the medicine is likely to be unsafe, ineffective or less beneficial than existing treatments (1).

In amyotrophic lateral sclerosis (ALS), disease onset manifests differently between young and adult patients (2–5). The mean age of adult-onset ALS varies between 40 and 63 years (6). An onset before the age of 25 (juvenile ALS) is rare (2,7) and before the age of 18 (pediatric ALS) is considered extremely unusual (3,4). The PDCO originally imposed a waiver substantiating the view that ALS is an adult-onset disease (8,9). However, in 2015 the waiver for ALS was revoked. The PDCO based their decision on new insights into the genetic origin of juvenile ALS, and the phenotypical similarities between adult-onset and juvenile-onset patients (10). As a consequence, pharmaceutical companies must now comply with a PIP to obtain marketing approval, or receive a waiver for their ALS drug developed for adults.

Whereas currently 14 trials in various clinical development phases are enrolling adult patients across Europe (11), no pediatric ALS trial has been conducted or planned. Given the absence of pediatric ALS trials to date, we aimed to (1) summarize potential future pediatric trials by exploring the status of submitted PIPs for ALS since 2015 and (2) investigate the feasibility for performing clinical studies in pediatric ALS patients in Europe by means of a questionnaire to clinical ALS centers. We sought to estimate the prevalence of pediatric ALS, potential recruitment rates at European ALS centers and gather opinions of European ALS experts on the revoked waiver for ALS.

## Methods

### Literature search PIPs

To determine the number and waiver status of PIPs since 2015, a literature search in the public database of the EMA (https://www.ema.europa.eu/en/medicines) was conducted. Documents were filtered by the following categories: “Humans,” “Pediatric Investigation Plans” and “decision date” from: “1 January 2015” to: “29 August 2020”. The keywords “Amyotrophic Lateral Sclerosis” and “Motor Neuron Disease” or “Motor Neurone Disease” were entered. The search criteria yielded a final selection of PIPs from which the following characteristics were gathered: PIP number, year of decision, drug, waived or waiver refused, reason for decision.

### Participants and questionnaire

Between August and December 2020, 58 experts with clinical ALS expertise were approached with a questionnaire by e-mail. All experts had an affiliation with a specialized clinical ALS center and with the European Network to Cure ALS (ENCALS: an active network consisting of ALS centers throughout Europe, www.encals.eu). To ensure a comprehensive online-questionnaire, all questions were reviewed and approved by the Executive Board of Treatment Research Initiative to Cure ALS (TRICALS, www.tricals.org) comprising neurologists from eight European countries (12).

The final questionnaire was divided into four sections. Written instructions were added per section for additional clarification. In the first section, the consent and the name of the center were collected. The second section included open quantitative questions per age subset on the total number of patients with ALS diagnosed at the center in the past 10 years. The age subsets were based on the denomination of the EMA: Adolescents (12–17 years); Children (2–11 years); Infants (1–23 months) and Neonates (0−27 days) (13). No identifiable patient data were collected. The third section entailed open questions to assemble quantitative data of the European ALS centers on recruitment capacity (per age subset) over a 5-year period. In addition, a multiple-choice question on potential recruitment hindrances was provided (multiple answers). The final section contained a statement on whether a waiver for ALS in specific age subsets should apply. The answers were followed by an open-ended question to collect the rationale behind the provided answer or a follow-up question consisting of multiple-choice answers. The questionnaire was implemented in Castor Electronic Data Capture (EDC) software.

### Data analysis

Unanswered or incomplete questions were excluded from the analysis. All quantitative data were compiled into descriptive statistics using frequencies (percentage) for categorical variables, and mean for continuous variables. Qualitative responses to open questions were merged and subsequently divided into themes.

## Results

### Literature search PIPs

Since the revocation of the waiver for ALS in 2015, four PIPs have been submitted by pharmaceutical companies to the EMA (Table 1). Two of the four PIPs received a waiver. One waiver was based on the disease not occurring in children as the drug, synthetic ribonucleic acid oligonucleotide (EMEA-002403-PIP01-18), aims to target a subset of patients with a superoxide dismutase 1 (*SOD1*) mutation (14). The second granted waiver for PIP EMEA-001266-PIP04-19 was based on no expected therapeutic effect of the compound, mastinib, in children (17). The other two PIPs (EMEA-001748-PIP02-18 and EMEA-002469-PIP01-18) for the compounds, arimoclomol citrate (15) and pyrimidinyl-aminopyridine dual leucine zipper kinase inhibitor (16), were not granted a waiver due to a potential therapeutic benefit in the pediatric population.

**Table 1:** Overview of submitted Pediatric Investigation Plans (PIPs) for ALS since 2015.

PIP number	Year of decision	Drug	Waived vs. waiver refused	Reason for the decision
EMEA-002403-PIP01-18	2018	Synthetic ribonucleic acid oligonucleotide directed against superoxide dismutase 1 (SOD1) messenger ribonucleic acid	Waived	The disease does not occur in the pediatric population. (14)
EMEA-001748-PIP02-18*	2018	Arimoclomol citrate	Waiver refused	The disease occurs in the pediatric population; The pediatric population has unmet need for treatment and may benefit from the medicinal product; The drug is not likely to be harmful; It may be feasible to conduct a clinical trial and resolve therapeutic needs. (15)
EMEA-002469-PIP01-18	2019	Pyrimidinyl-aminopyridine dual leucine zipper kinase inhibitor	Waiver refused	The disease occurs in the pediatric population; The pediatric population has unmet need for treatment and may benefit from the medicinal product; The drug is not likely to be harmful; It may be feasible to conduct a clinical trial and resolve therapeutic needs. (16)
EMEA-001266-PIP04-19	2020	Mastinib	Waived	No therapeutic benefit to be expected for the pediatric population. (17)

*During the preparation of this manuscript, the pivotal trial with Arimoclomol citrate (NCT03491462) did not meet its primary and secondary endpoints.

### Questionnaire – response rate

We invited 58 ALS centers from 22 European countries to answer the questionnaire. We received a response from 49 centers (response rate 84.5%) located in 19 different countries. Of these 49 responders, three sites did not answer all questions.

### Questionnaire – prevalence and recruitment potentials

To estimate the prevalence of pediatric ALS in Europe, all participating ALS centers were asked to provide the number of patients diagnosed at their center during the past ten years. In total, 44,858 ALS patients had been diagnosed in the past ten years in 46 centers, of whom 0.087% (*n* = 39) were under the age of 18 (Table 2). The ALS centers (*n* = 47) estimated that a total of 26 pediatric patients could be recruited over a timeframe of five years (Table 3). Factors that potentially hinder recruitment were divided into eight different categories. Almost half of the ALS centers (45.7%) reported that pediatric ALS is rare and that this would hinder patient recruitment (Table 4).

**Table 2:** Patients with ALS diagnosed in the past 10 years by 46 European ALS centers.

	No. of patients diagnosed in past 10 years	
Age subsets	N	Prevalence %
All ages	44,858	–
Adolescents (12–17 years)	32	0.07%
Children (2–11 years)	7	0.02%
Infants (1–23 months)	0	0.00%
Neonates (0−27 days)	0	0.00%

**Table 3:** Expected recruitment by 47 European ALS centers.

	Expected recruitment within 5 years	
Age subsets	N	Mean per 10 sites
Adolescents (12–17 years)	18	3.83
Children (2–11 years)	8	1.70
Infants (1–23 months)	0	0.00
Neonates (0−27 days)	0	0.00

**Table 4:** Factors that might hinder recruitment of pediatric patients with ALS (multiple answers possible).

Characteristic	Responders (*n* = 46) answering yes *n* (%)
Disease is too rare and low occurrence	21 (45.7)
Diagnostic delay	13 (28.3)
Patients are seen by pediatricians	9 (19.6)
Parental involvement	9 (19.6)
Lack of expertise at the center	9 (19.6)
Excessive demand on few patients due to multiple recruiting trials	8 (17.4)
Ethical reasons	5 (10.9)
Study burden	5 (10.9)

### Questionnaire – waiver statement

**Figure 1:**
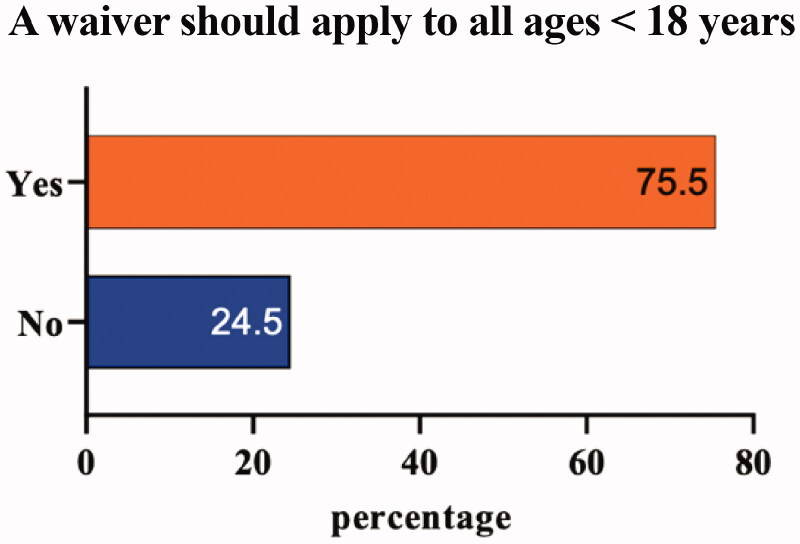
ALS experts’ opinions (yes or no) on whether a waiver should apply to patients with ALS below the age of 18, *n* = 49.

Figure 1 summarizes the responses of ALS experts (*n* = 49) to whether a general waiver should be applied for patients with ALS under 18; while 37 centers (75.5%) agreed that a waiver should be applied for all ages <18 years, 12 centers (24.5%) did not. Those not agreeing specified that clinical trials should be carried out due to the need of a treatment (*n* = 4, 36.4%). The main reason given by ALS experts (*n* = 35, 94.1%) that a waiver should be applied was based on pediatric patients being too rare to enroll in clinical trials (Table 5).

**Table 5:** Reasons for applying a waiver to all ages below 18 years (multiple answers possible).

Yes (*n* = 35)	*n* (%)
Too rare to enroll sufficient patients	35 (94.1)
Phenotypically different from adult-onset ALS	16 (43.2)
Rarely sporadic but primarily familial	13 (35.1)
No* (*n* = 11)	***n* (%)**
Clinical trials are needed for the treatment of pediatric ALS	4 (36.4)
Waiver should apply for < 11 years as there are no patients under 11 years of age	3 (27.3)
Waiver should apply for < 11 years due to age-related difference in safety and efficacy	2 (18.2)
Pediatric ALS is a different disease than adult-onset ALS	2 (18.2)

*One reason was excluded as ‘not applicable’ was answered.

## Discussion

In this study, we aimed to (1) summarize potential future pediatric studies and (2) assess the feasibility of pediatric trials in Europe. Two PIPs received the decision of the PDCO that the drug interventions were expected to be beneficial for pediatric patients and that a pediatric study is, therefore, required. With an estimated prevalence of pediatric ALS being less than 0.1% of all patients diagnosed with ALS, enrolling sufficient numbers in these studies is an undoubted challenge. Centers estimated a recruitment rate of 26 patients within 5 years. Conducting a pediatric clinical trial would require extensive recruitment periods, design challenges and high operational costs, while ultimately providing little information on the drug’s safety and/or efficacy.

Presuming a positive outcome of a late-phase clinical trial in the adult ALS population in Europe, the first clinical studies may commence in the coming year(s) for pediatric ALS patients. However, we find a placebo-controlled clinical trial to be untenable. Less than 0.1% of all patients with ALS diagnosed in the past 10 years by the 46 European centers were under 18. When we apply this percentage to the prevalence of ALS (8 per 100,000 persons) (18), around 0.008 cases per 100,000 persons might be affected by pediatric ALS in Europe. These numbers would suggest that pediatric ALS classifies as an ultra-rare disorder (≤ 1 per 50,000) (19).

To determine the resources for any trial based on our estimated recruitment rate (3.83 adolescent patients/10 sites/5 years), we assumed a sample size of ten adolescent patients. The trial would require 30 sites to enroll ten patients within a 5-year period. This estimation does not take into account potential screening failures, concomitant other diseases prohibiting participation, withdrawals or other competing trials (11). Also, in rare disease trials, each clinical phase is often twice as long due to lack of information on disease prevalence and incidence, eligible patients and difficulties in timely patient recruitment (20) and adds additional operational costs (21). In addition, pediatric trials in general are accompanied by various ethical concerns *i.e.,* determining the scientific necessity, welfare of the children, risk vs. benefits, and legal implications (*i.e.,* extensive ethical approval processes and refusal of parents to give consent) (22).

There are important implications regarding trial design for a small population and sample sizes as often seen in rare disease trials (23). As required by the EMA, a PIP should describe how efficacy and (long-term) safety can be measured in the pediatric population. To determine efficacy (e.g. slowing of disease progression or improvement in survival time) in a patient population, it is not uncommon to require sample sizes of at least 50 patients to detect large effect sizes (11). These numbers are virtually impossible for a rare disease such as pediatric ALS, and quantifying efficacy would be difficult unless the treatment benefit is considerable (e.g. complete stop of disease progression or reversal of symptoms). While a trial based on safety endpoints may require fewer participants, it is not clear whether safety data originating from a few patients will provide sufficient information to allow definite conclusions to be drawn (e.g. uncommon adverse events can be easily missed by chance). To measure survival time or disease progression in patients with pediatric ALS, the duration of the follow-up period will require a longer period than in adults to detect changes over time. Furthermore, it is questionable if ALS endpoints and trial methodologies can be adopted to pediatric ALS studies due to the differences in phenotype and clinical characteristics between children and adult patients.

There are differences between regulatory landscapes across continents compared to Europe. Regulatory authorities, for example in Canada and Japan, do not legally require pharmaceutical companies to include pediatric study plans for adult drug development (24,25). The U.S. Food and Drug Administration (FDA) does have a similar legislation as Europe; however, a waiver is granted for pediatric ALS studies based on the rarity of the disease, limited occurrence in children and studies being highly unfeasible or even impossible (26). It is recognized that the PDCO’s scientific rational to revoke the waiver for ALS was based on novel genetic insights with regard to juvenile ALS and the apparent similarity in phenotypical and clinical representations between juvenile and adult onset ALS (10). However, this in turn raises the question whether the revocation of the waiver by the PDCO was based on valid scientific arguments. A clear definition of pediatric ALS is lacking and there is no consensus among ALS experts on the disease entity. Indeed, there is considerable evidence that pediatric and juvenile ALS are separate entities that are distinct from adult-onset ALS (2–5). Patients diagnosed with ALS below the age of 18 are characterized by a slower disease progression, longer survival time and are more likely to exhibit underlying genetic variants of major effect (2–4). By contrast, adults with ALS exhibit 5–6 (27) steps in disease development, which reduce to 2-4 in those with major disease-causing variants (28).

There are resemblances between pediatric- and adult-onset ALS. For example, P525L mutation in FUS can cause disease pathology in both pediatric, juvenile (4) and adultpatients (29). Notwithstanding, it is likely that a pathology will be different between infants with a developing neuroaxis, young adults with a maturing neuroaxis and mid-life individuals with established pathways and networks.

An alternative approach would be to recognize that pediatric ALS is an ultra rare disease, and that there may be alternate approaches to provide access to new therapeutics. As we have identified a cohort of patients with pediatric ALS, there are multiple options to gather information on potential (new) drugs, e.g. by extrapolating data or by focusing on pharmacokinetics and/or pharmacodynamics parameters in a small subset. Additionally, considering the genetic cause of pediatric ALS, a genotype-targeted approach may yield more positive outcomes for pediatric ALS, as currently being investigated in familial adult-onset ALS (30–32). Genotype targeted therapies have been proven successful in the treatment of spinal muscular atrophy (SMA) (33). Best practices of genotype targeted treatments, as seen in SMA trials, could potentially be applied to future pediatric ALS trial. Trial success may be achieved by involving family members at an early stage and during the trial, using websites with updated information on the trial, setting up of large-scale databases to (pre-) identify patients, offering asymmetric randomization and an open label extension (34).

In this study we did not gather detailed clinical and genetic patient data to ensure that patients remain unidentifiable. Gathering clinical and genetic patient data in a secure manner for future clinical trials will be essential for personalized treatment approaches. A strength of this study is that we were able to identify patients with pediatric ALS based on data provided by ALS experts across Europe. Our finding of 39 patients in the past 10 years was higher than the number in a recent publication by Pincher-Martel et al. (4). The authors reported 29 pediatric cases. Based on their collected data, we observed that 30% of identified cases by the authors were European patients, i.e., nine European patients over a timeframe of ∼15 years. The discrepancy between our reported prevalence compared to the number of patients identified by Pincher-Martel et al. (2020) could be explained by patients not being captured in case reports.

We unreservedly support the PDCO and their endeavors to improve access to medicines for children. However, we respectfully question whether a PIP is the best approach to achieve this. Pediatric ALS studies will require lengthy recruitment periods, increased operational costs, ethical and legal implications and implications in trial design (e.g.*,* limited statistical power and efficacy/safety evidence). Given the absence of consensus on pediatric ALS as a disease entity, the extremely low prevalence of ALS in the pediatric population and the potential difficulties in recruiting sufficient patients, appropriately powered clinical trials are simply not feasible.

For these reasons, and based on the responses of ALS experts, we suggest the decision whether or not to grant a waiver should be reconsidered for patients with ALS under the age of 18. Rather than exhausting financial resources for mandated studies in a scarce population, companies and investigators could contribute by investing resources in the understanding of pediatric ALS and to advance targeted treatment approaches.

## References

[1] The European Parliament and The Council of the European Union. Regulation No 1901/2006 of the European Parliament and of the Council on Medicinal Products for Paediatric Use and Amending Regulation. Off J Eur Union 2006;378:1–19.

[2] Sabatelli M, Madia F, Conte A, Luigetti M, Zollino M, Mancuso I, et al. Natural history of young-adult amyotrophic lateral sclerosis. Neurology 2008;71:876–81. 18596241 10.1212/01.wnl.0000312378.94737.45

[3] Gouveia LO, de Carvalho M. Young-onset sporadic amyotrophic lateral sclerosis: a distinct nosological entity? Amyotroph Lateral Scler. 2007;8:323–7. 17852021 10.1080/17482960701553956

[4] Picher-Martel V, Brunet F, Dupré N, Chrestian N. The occurrence of FUS mutations in pediatric amyotrophic lateral sclerosis: a case report and review of the literature. J Child Neurol. 2020;35:556–62. 32281455 10.1177/0883073820915099

[5] Finsterer J, Burgunder JM. Recent progress in the genetics of motor neuron disease. Eur J Med Genet. 2014;57:103–12. 24503148 10.1016/j.ejmg.2014.01.002

[6] Logroscino G, Traynor BJ, Hardiman O, Chió A, Mitchell D, Swingler RJ, et al. Incidence of amyotrophic lateral sclerosis in Europe. J Neurol Neurosurg Psychiatry. 2010;81:385–90. 19710046 10.1136/jnnp.2009.183525 PMC2850819

[7] Turner MR, Barnwell J, Al-Chalabi A, Eisen A. Young-onset amyotrophic lateral sclerosis: Historical and other observations. Brain. 2012;135:2883–91. 22661746 10.1093/brain/aws144

[8] European Medicines Agency. Decision Class Waiver on conditions - granting, review, revocation. 2011. Available at: https://www.ema.europa.eu/en/documents/other/cw/1/2011-ema-decision-19-december-2011-class-waiver-conditions_en.pdf. Accessed December 1, 2020.

[9] European Medicines Agency. Decision Class Waiver on conditions - granting, review, revocation. 2010. Available at: www.ema.europa.eu/en/documents/other/p/63/2010-ema-decision-20-december-2010-class-waiver-conditions_en.pdf. Accessed December 1, 2020.

[10] European Medicines Agency. Summary Report on the review of the list of granted Class Waivers (EMA/PDCO/178231/2015EMA/PDCO). 2015;44(0).

[11] van Eijk RPA, Kliest T, van den Berg LH. Current trends in the clinical trial landscape for amyotrophic lateral sclerosis. Curr Opin Neurol. 2020;33:655–61. 32796282 10.1097/WCO.0000000000000861

[12] van Eijk RPA, Kliest T, McDermott CJ, Roes KCB, Van Damme P, Chio A, et al. TRICALS: creating a highway toward a cure. Amyotroph Lateral Scler Front Degener 2020;1–6. 10.1080/21678421.2020.1788092 32643415

[13] The European Parliament and The Council of the European Union. Guideline on good pharmacovigilance practices (GVP).. 2012;(February):1–47. Available at: http://www.jfda.jo/Download/JPC/TheGoodPharmacovigilancePracticev2.pdf. Accessed 1 December 2020.

[14] Opinion of the Paediatric Committee on the granting of a product-specific waiver EMEA-002403-PIP01-18. Available at: https://www.ema.europa.eu/en/documents/pip-decision/p/0337/2018-ema-decision-8-october-2018-granting-product-specific-waiver-synthetic-ribonucleic-acid_en.pdf. Accessed August 29, 2020.

[15] European Medicines Agency. Opinion of the Paediatric Committee on the granting of a product-specific waiver and on the refusal of a product- specific waiver EMEA-001748-PIP02-18. Available at: https://www.ema.europa.eu/en/documents/pip-decision/p/0317/2018-ema-decision-12-september-2018-granting-product-specific-waiver-arimoclomol-citrate-condition_en.pdf. Accessed August 29, 2020.

[16] European Medicines Agency. Opinion of the Paediatric Committee on the refusal of a product-specific waiver EMEA-002469-PIP01-18. Available at: https://www.ema.europa.eu/en/documents/pip-decision/p/0099/2019-ema-decision-22-march-2019-refusal-product-specific-waiver-pyrimidinyl-aminopyridine-dual_en.pdf. Accessed August 29, 2020.

[17] European Medicines Agency. Opinion of the Paediatric Committee on the granting of a product-specific waiver EMEA-001266-PIP04-19. Available at: https://www.ema.europa.eu/en/documents/pip-decision/p/0001/2020-ema-decision-3-january-2020-granting-product-specific-waiver-masitinib-mesylate-emea-001266_en.pdf. Accessed August 29, 2020.

[18] Hardiman O, Al-Chalabi A, Brayne C, Beghi E, Van Den Berg LH, Chio A, et al. The changing picture of amyotrophic lateral sclerosis: lessons from European registers. J Neurol Neurosurg Psychiatry. 2017;88:557–63. 28285264 10.1136/jnnp-2016-314495

[19] The European Parliament and The Council of the European Union. Clinical trials regulation. Off J Eur Union 2014;2014:55–7.

[20] Jayasundara K, Hollis A, Krahn M, Mamdani M, Hoch JS, Grootendorst P. Estimating the clinical cost of drug development for orphan versus non-orphan drugs. Orphanet J Rare Dis. 2019;14:12. 30630499 10.1186/s13023-018-0990-4 PMC6327525

[21] Berdud M, Drummond M, Towse A. Establishing a reasonable price for an orphan drug. Cost Eff Resour Alloc. 2020;18:1–18. 32908456 10.1186/s12962-020-00223-x PMC7472708

[22] Anzelewicz S, Garnier H, Rangaswami A, Czauderna P. Cultural, geographical and ethical questions when looking to enroll pediatric patients in rare disease clinical trials. Expert Opin Orphan Drugs. 2017;5:613–21.

[23] Day S, Jonker AH, Lau LPL, Hilgers RD, Irony I, Larsson K, et al. Recommendations for the design of small population clinical trials. Orphanet J Rare Dis. 2018;13:1–9. 30400970 10.1186/s13023-018-0931-2 PMC6219020

[24] Hepburn CM, Gilpin A, Autmizguine J, Denburg A, Dupuis LL, Finkelstein Y, et al. Improving paediatric medications: a prescription for Canadian children and youth. Paediatr Child Health. 2019;24:333–9. 31379437 10.1093/pch/pxz079 PMC6656949

[25] Saitou H, Nakatani D, Myoui A, Kubota T, Ozono K. Pediatric drug development in Japan: current issues and perspectives. Clin Pediatr Endocrinol. 2020;29:1–7. 32029968 10.1297/cpe.29.1 PMC6958521

[26] U.S. Food and Drug Administration. List of diseases for which FDA automatically grants a full waiver of pediatric studies. Available at: https://www.fda.gov/media/101440/download. Accessed December 16, 2021.

[27] Al-Chalabi A, Calvo A, Chio A, Colville S, Ellis CM, Hardiman O, et al. Analysis of amyotrophic lateral sclerosis as a multistep process: a population-based modelling study. Lancet Neurol. 2014;13:1108–13. 25300936 10.1016/S1474-4422(14)70219-4 PMC4197338

[28] Chiò A, Mazzini L, D’Alfonso S, Corrado L, Canosa A, Moglia C, et al. The multistep hypothesis of ALS revisited: the role of genetic mutations. Neurology 2018;91:e635–42. 30045958 10.1212/WNL.0000000000005996 PMC6105040

[29] Andersen P, Al-Chalabi A. Clinical genetics of amyotrophic lateral sclerosis: what do we really know? Nat Rev Neurol. 2011;7:603–15. 21989245 10.1038/nrneurol.2011.150

[30] Chiò A, Mazzini L, Mora G. Disease-modifying therapies in amyotrophic lateral sclerosis. Neuropharmacology 2020;167:107986. 32062193 10.1016/j.neuropharm.2020.107986

[31] Van Eijk RPA, Jones AR, Sproviero W, Shatunov A, Shaw PJ, Leigh PN, et al. Meta-analysis of pharmacogenetic interactions in amyotrophic lateral sclerosis clinical trials. Neurology 2017;89:1915–22. 28978660 10.1212/WNL.0000000000004606 PMC5664299

[32] Armon C, Hardiman O. The beginning of precision medicine in ALS? Treatment to fit the genes. Neurology 2017;89:1850–1. 28978653 10.1212/WNL.0000000000004612

[33] Finkel RS, Mercuri E, Darras BT, Connolly AM, Kuntz NL, Kirschner J, et al. Nusinersen versus sham control in infantile-onset spinal muscular atrophy. N Engl J Med. 2017;377:1723–32. 29091570 10.1056/NEJMoa1702752

[34] Iannaccone ST, Nelson LL. (2017). Spinal muscular atrophy clinical trials. in Spinal Muscul Atrophy (pp. 423-428)Elsevier Inc.

